# Quality of systematic reviews on the treatment of vesiculobullous skin diseases. A meta-epidemiological study^⋆^

**DOI:** 10.1016/j.abd.2023.06.003

**Published:** 2023-11-18

**Authors:** Kamilla Mayr Martins Sá, Juliana Cavaleiro Rodrigues, Lígia Borges da Silva, Giovanna Marcılio Santos, Mileny Esbravatti Stephano Colovati, Ana Luiza Cabrera Martimbianco

**Affiliations:** a Department of Medicine, Universidade Metropolitana de Santos, Santos, SP, Brazil; b Postgraduate Program in Health and Environment, Department of Medicine, Universidade Metropolitana de Santos, Santos, SP, Brazil; c Health Technology Assessment Center, Hospital Sírio-Libanês, São Paulo, SP, Brazil

**Keywords:** Bias, Evidence-based medicine, Skin diseases, vesiculobullous, Systematic reviews as a topic

## Abstract

**Background:**

Systematic reviews of Randomized Controlled Trials (RCTs) are considered high-level evidence to support a decision on therapeutic interventions, and their methodological quality is essential to provide reliable and applicable results.

**Objective:**

This meta-epidemiological study aimed to map and critically appraise systematic reviews assessing treatments for vesiculobullous skin diseases.

**Methods:**

We conducted a comprehensive search strategy on MEDLINE (via Pubmed) in December 2022 without restrictions to find systematic reviews evaluating pharmacological interventions for vesiculobullous skin diseases. The methodological quality was assessed using the AMSTAR-2 tool, and additional information was extracted. We identified nine systematic reviews published between 2002 and 2021, seven assessing pemphigus.

**Results:**

According to the AMSTAR-2 tool, 55.6% were classified as critically low quality, 22.2% as moderate quality, 11.1% as low and 11.1% as high quality. No review assessed the certainty of the evidence (GRADE); 86% of pemphigus reviews had at least two overlapping RCTs. There were some limitations regarding methodological flaws and the AMSTAR-2 tool use

**Conclusions:**

These findings reveal a frail methodological quality of systematic reviews about vesiculobullous diseases treatment that may impact the results. Therefore, methodological rigor is mandatory for future systematic reviews to avoid duplication of effort and increase the certainty of the evidence supporting decision-making.

## Introduction

Systematic reviews of Randomized Controlled Trials (RCTs) are studies designed to identify, summarise and critically appraise the best available evidence on therapeutic interventions and provide allowance to the decision regarding patient care and health policy.^1^, ^2^ This study design aims to avoid the unnecessary and redundant production of primary studies, provides quick answers to health questions, and can combine results from similar studies in meta-analysis, increasing the statistical power and confidence in estimated effects.^1^ By applying adequate methodological recommendations for developing a systematic review of intervention, a careful evaluation of the included RCTs is conducted, directly implying the reliability of the evidence through the results obtained since RCTs are the most suitable primary study design to answer a clinical treatment question. Thus, the applicability of the results on the efficacy and safety of treatments in clinical practice can help health professionals, policymakers, patients, and guideline producers.^1^

Rigorously developed evidence in dermatology has increased over time, primarily due to concerns about the impact of methodological flaws on the estimated effects of interventions evaluated in clinical trials. As a result of the numerous publications of RCTs on dermatological treatments, a growing number of systematic reviews have been published in this area, and the appropriate methodological recommendations for preparing and conducting a systematic review, as recommended by Cochrane, are not always followed by the authors, leading to systematic errors and biased results, most of the time overestimating the evaluated treatment.^1^, ^3^, ^4^

Regarding that, in the last years, meta-epidemiological studies have been conducted to evaluate the impact of some methodological limitations of clinical studies on the observed results. Additionally, these meta-research studies verify the methodological quality to support future research precluding the occurrence of the same biases and drawing reliable evidence to establish decision-making processes in healthcare. The unit of analysis in a meta-epidemiological study is individual studies and not patients, as in traditional clinical research.^5^, ^6^

The treatment of dermatoses, such as vesiculobullous diseases, has been investigated by several studies. These clinical skin conditions present the appearance of vesicles or blisters as primary clinical manifestations, directly affecting the patient's quality of life. These are affections of variable etiology and may result from infection, genetic or metabolic alterations, drug hypersensitivity, and autoimmune conditions.^7^, ^8^, ^9^ Since the emergence of corticosteroid therapy, these patients have had access to treatment resources, which could be associated with other drug classes, such as immunosuppressants.^10^ The severity of the disease and the need for effective and safe treatments reinforce the requirement for clinical studies with high methodological quality. Thereby, the purpose of this meta-epidemiological study is to map and critically appraise systematic reviews that assessed pharmacological treatments for vesiculobullous diseases.

## Methods

This meta-epidemiological study follows the recommendations proposed by Murad et al. 2017 for reporting meta-epidemiological studies,^4^ in addition to the pertinent items of the Preferred Reporting Items for Systematic Reviews and Meta-Analyses (PRISMA 2020).^11^

### Criteria for including studies

#### Types of studies

We considered systematic reviews that include only Randomized Clinical Trials (RCTs) on interventions for treating vesiculobullous skin diseases, such as acantholysis, blister, dermatitis herpetiformis, epidermolysis bullosa, erythema multiforme, Stevens-Johnson syndrome, bullous impetigo, hydroa vacciniforme, linear IgA bullous dermatosis, pemphigoid and pemphigus. Protocols or earlier versions of the same systematic review were not included. Articles that did not have the term systematic review in the title or body of the text were not considered. Systematic reviews available only as abstracts, incomplete formats, or in progress were not considered.

### Data analysis

1 Characteristics of included systematic reviews: year of publication, number of primary included RCTs, type of vesiculobullous disease, types of pharmacological interventions.
2 Methodological quality of the included systematic reviews according to the items of the AMSTAR-2 tool.^12^
3 Adequacy of search strategies report by applying the following aspects (complete search date presented (month/day/year), search strategy presented (Mesh terms used), additional hand searches conducted, restrictions applied (language and date), and databases searched.^13^
4 Number of systematic reviews that assessed the certainty of the evidence using the Grading of Recommendations Assessment, Development, and Evaluation (GRADE) approach.^14^ It is worth mentioning that the GRADE approach was implemented in 2013, and systematic reviews published before that certainly did not use this tool.
5 Number of systematic reviews that identify and report 'awaiting classification studies' (a study found in the search does not provide enough information to analyze compliance with the eligibility criteria, e.g. full text unavailable). This study remains in a state of awaiting evaluation until additional information is obtained), and ongoing studies (randomized clinical trials that are still in progress, registered in databases such as Clinical Trials.gov, that may contribute to the results when finalized).^13^
6 To verify how many RCTs were included in more than one systematic review to identify studies overlapping.

### Search for studies

Systematic reviews were identified from the Medical Literature Analysis and Retrieval System Online (MEDLINE, via PubMed) database (December 01, 2022), with filtering limits for study design (systematic review). No restrictions on publication date or language will be applied. Since it was a meta-epidemiological study and the authors are looking for a sample of systematic reviews on the treatment of vesiculobullous skin diseases, we considered the search on MEDLINE (via Pubmed) sufficient. The search strategy is detailed in Table 1.

**Table 1:** Search strategies for the MEDLINE database (via PubMed).

Search strategy
(“Skin Diseases, Vesiculobullous” [Mesh] OR (Skin Disease, Vesiculobullous) AND (Vesiculobullous Skin Disease) OR (Vesiculobullous Skin Diseases) OR (Vesiculobullous Dermatoses) OR (Dermatoses, Vesiculobullous) OR (Skin Diseases, Bullous) OR (Bullous Skin Disease) OR (Skin Disease, Bullous) OR (Bullous Skin Diseases) OR (Bullous Dermatoses) OR (Dermatoses, Bullous) OR (Vesicular Skin Diseases) OR (Skin Disease, Vesicular) OR (Vesicular Skin Disease) OR (Skin Diseases, Vesicular) OR (Pustular Dermatosis, Subcorneal) OR (Dermatoses, Subcorneal Pustular) OR (Dermatosis, Subcorneal Pustular) OR (Pustular Dermatoses, Subcorneal) OR (Subcorneal Pustular Dermatoses) OR (Sneddon-Wilkinson Disease) OR (Sneddon Wilkinson Disease) OR (Subcorneal Pustular Dermatosis)) AND (systematic [sb])

### Selection of studies

The study selection process was conducted using the Rayyan platform.^15^ Two reviewers independently evaluate all titles and abstracts retrieved by the search strategies. First, the potentially eligible studies were analyzed in full text to confirm eligibility. Inconsistencies between reviewers were solved by consulting a third reviewer. Studies excluded after the second step were presented in the 'excluded studies table' along with the reasons for each exclusion.

### Data extraction

The procedures for data extraction were carried out by an independent pair of reviewers using a pre-established data sheet. Inconsistencies during this process were solved by consulting a third reviewer.

### Methodological quality assessment of included systematic reviews

The methodological quality of the reviews was assessed by a score based on the AMSTAR-2 tool,^12^ which includes 16 items addressing (1) Whether the research questions and inclusion criteria of the review include the PICO components; (2) Whether there was an a priori protocol; (3) Whether there are justifications for the selection of the study design; (4) What the search strategies were; (5) Whether duplicate studies were selected; (6) Whether duplicate data were extracted; (7) Whether excluded studies and the reasons for exclusion were presented; (8) What were the characteristics of the included studies; (9) What were the methods for assessing the risk of bias of the included studies; (10) Whether the funding source of the included studies was reported; (11) Whether the methods for combining the results were adequate; (12) Whether there was an assessment of the impact of the risk of bias on the results of the meta-analysis; (13) Whether the risk of bias was considered in the interpretation and discussion of the results; (14) Whether there is discussion and explanation of heterogeneity; (15) Whether there was investigation of publication bias; and (16) Whether there was a declaration of conflict of interest of the review authors. Seven of these items are considered critical (items 1, 4, 7, 9, 11, 13 and 15), and in the end, the review is classified as having one of the following degrees of confidence: critically low (more than one critical flaw), low (one critical flaw), moderate (more than one non-critical flaw) and high (none or one non-critical flaw). The overall confidence will be generated using the checklist tool available on the AMSTAR-2 website (http://amstar.ca/Amstar_Checklist.ph). Judgment of the AMSTAR-2 items of the included reviews was applied in each included systematic review by two authors independently, and a third author will resolve disagreements.

### Data analysis and presentation

Descriptive statistics were presented with absolute and relative frequencies (in percentages) and summarized in graphs and tables. The analyses will be carried out using Microsoft Excel®.

## Results

### Search results

**Figure 1:**
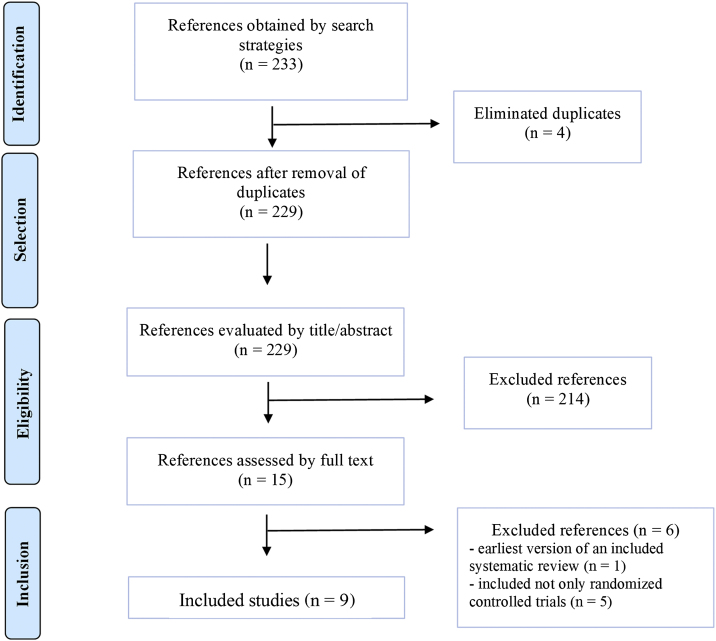
Flowchart of the study selection process.

The electronic search retrieved 233 references. After removing four duplicates, 229 titles and abstracts were screened, and 214 did not meet the eligibility criteria. Thus, 15 studies were analyzed in full text, and six were excluded: one was the earliest version of an included systematic review^16^ and five because they had not only RCTs.^17^, ^18^, ^19^, ^20^, ^21^ Therefore, nine systematic reviews^22^, ^23^, ^24^, ^25^, ^26^, ^27^, ^28^, ^29^, ^30^ were included (Fig. 1).

### Characteristics of the included systematic reviews

The nine systematic reviews were published between 2002 and 2021 and assessed only Randomized Clinical Trials (RCTs) as the primary study, including between one and 20 RCTs. In addition, three reviews (33.3%)^25^, ^27^, ^28^ are Cochrane reviews. Table 2 shows the main characteristics of the included systematic reviews.

**Table 2:** Characteristics of the included systematic reviews.

Systematic reviews	Included RCT (n)	Population	Interventions and comparators	Outcome assessed by the systematic reviews	Financial sources
Asilian 2021^22^	3	Bullous pemphigoid	Methylprednisolone	Mortality	No funding sources reported.
			Azathioprine	Disease control	
			Dapsone	Adverse Events	
			Doxycycline	Time to complete response	
			Prednisolone		
			Intravenous immunoglobulin		
Atzmony 2014^23^	20	Pemphigus vulgaris, pemphigus foliaceus	Azathioprine	Complete response	No funding sources reported.
			Mycophenolate mofetil	Mean total cumulative glucocorticoid dose	
			Cyclophosphamide		
			Intravenous immunoglobulin	Mortality	
				Disease control	
				Remission	
				Relapse	
				Withdrawal due to adverse events	
Atzmony 2015^24^	10	Pemphigus vulgaris, pemphigus foliaceus	Glucocorticoid	Remission	No funding sources reported.
			Azathioprine	Disease control	
			Mycophenolate mofetil	Relapse	
			Cyclophosphamide	Cumulative glucocorticoid dose	
			Cyclosporine	Withdrawal due to adverse events	
			Intravenous immunoglobulin plasma exchange	Mortality	
			Infliximab		
Kirtschig 2010^25^	10	Bullous pemphigoid	Prednisolone	Disease control	No funding sources reported.
			Azathioprine	Mortality	
			Plasma exchange	Overall improvement	
			Mycophenolate mofetil, tetracycline	Several adverse events	
			Nicotinamide	Quality of life	
			Chinese traditional medicine	Remission	
				Systemic infection	
				Organ failure	
				Allergic reactions	
Garcia-Doval 2013^26^	7	Inherited forms of epidermolysis bullosa	Tetracyclines	Overall improvement	No funding sources reported.
			Oxytetracycline	Prevention of new lesions	
			Aluminium chloride hexahydrate solution	Pain and pruritus	
				Quality of life	
			Bufexamac cream	Expression of COL7A1 mRNA, type 7 collage	
			Phenytoin	Anchoring fibrils	
			Cultured allogeneic fibroblasts	Number and characteristics of lesions	
			Trimethoprim		
				Patient perception	
				Number of infection	
				Emergence of resistant bacteria	
Majumdar 2002^27^	1	Toxic epidermal necrolysis	Thalidomide	Mortality	No funding sources reported.
				Quality of life	
				Pain during acute episode	
				Loss of total body surface area	
				Serious infection	
				Renal failure	
				Length of hospital stay	
				Bone marrow toxicity	
Martin 2009^28^	11	Pemphigus vulgaris and pemphigus foliaceus	Prednisolone	Remission	No funding sources reported.
			Dexamethasone	Mortality	
			Azathioprine	Disease control	
			Cyclophosphamide	Relapse	
			Cyclosporine	Change in pemphigus severity score	
			Dapsone		
			Mycophenolate	Time to disease control	
			Plasma exchange	Cumulative glucocorticoid dose	
			Topical epidermal growth factor	Reduction of serum antibody titres	
			Traditional Chinese medicine	Adverse events	
				Quality of life	
Singh 2011^29^	19	Pemphigus vulgaris, pemphigus foliaceus, and bullous pemphigoid	Prednisolone	Adverse Events	No funding sources reported.
			Methylprednisolone	Overall improvement	
			Dexamethasone	Remission	
			Cyclophosphamide		
			Dapsone		
			Intravenous immunoglobulin		
			Mycophenolate mofetil		
			Cyclosporine		
			Azathioprine		
Zhao 2015^30^	18	Pemphigus vulgaris	Mycophenolate mofetil	Mortality	No funding sources reported.
			Azathioprine	Disease control	
			Intravenous immunoglobulins	Relapse	
			Sulfasalazine	Withdrawal due to adverse events	
			Pentoxifylline		
			Infliximab		
			Epidermal growth factor		
			Pimecrolimus		

n, Number of included Randomized Clinical Trials (RCT).

The following interventions were analyzed for treating vesiculobullous skin disease: corticosteroids, cyclosporine, intravenous immunoglobulin, plasma exchange, dapsone, doxycycline, tetracycline, mycophenolate mofetil, cyclophosphamide, tetracyclines, oxytetracycline, aluminum chloride hexahydrate solution, bufexamac cream, phenytoin, epidermal growth factor, pimecrolimus, cultured allogeneic fibroblasts, trimethoprim and thalidomide.

Of the nine systematic reviews analyzed, 55.6% (5/7)^23^, ^24^, ^28^, ^29^, ^30^ studied patients with bullous pemphigus (pemphigus foliaceus and/or vulgaris), 22.2% (2/9)^22^, ^25^ with bullous pemphigoid; 11.1% (1/9)^26^ with epidermolysis bullosa and 11.1% (1/9)^27^ with toxic epidermal necrolysis.

### Methodological quality assessment

**Figure 2:**
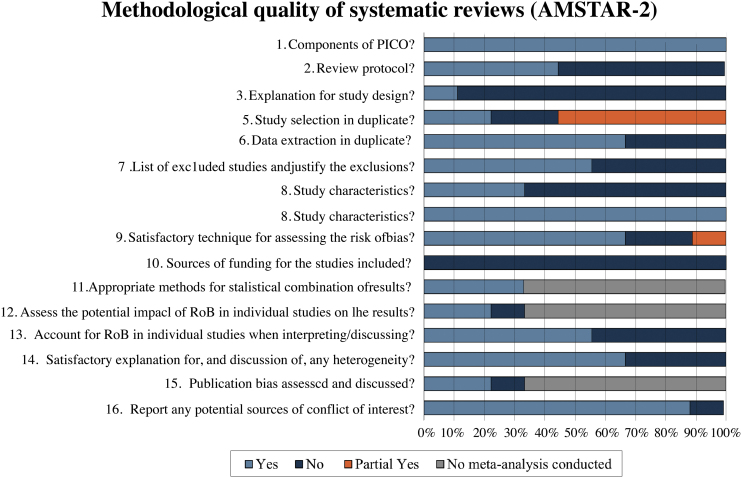
Methodological quality assessment through AMSTAR-2.

The methodological quality of the reviews was analyzed using the AMSTAR-2 tool. In the assessment, 55.6% (5/9)^22^, ^23^, ^26^, ^29^^,^^30^ of reviews were classified as critically low quality, 22.2% (2/9)^25^, ^27^ as moderate quality, 11.1% (1/9)^24^ as low quality and 11.1% (1/9)^28^ as high quality. Fig. 2 describes a summary of findings separating AMSTAR-2 assessment by items.

Systematic reviews classified as critically low quality^22^, ^23^, ^26^, ^29^^,^^30^ presented a negative response in almost the same items. None of them reported a review protocol registration (item 2), an explanation of the primary study design selection (item 3), a list of excluded studies with reasons (item 7), and funding sources of each included RCT (item 10). Of these reviews, 40% (2/5)^22^, ^23^ did not present a comprehensive search strategy (item 4); 60% (3/5)^26^, ^29^, ^30^ did not perform study selection and data extraction by two independent authors (items 5 and 6); and 40% (2/5)^22^, ^30^ did not use a satisfactory technique for assessing the Risk of Bias (RoB) in individual RCTs (item 9). The only study in this classification that performed meta-analysis^23^ did not evaluate the impact of risk of bias of individual studies in the meta-analysis (item 12) and did not perform publication bias investigation (item 15). Four of the five reviews^22^, ^23^, ^29^, ^30^ (80%) did not account for RoB in individual studies when interpreting/discussing the results of the review (item 13), and three^22^, ^29^, ^30^ (60%) did not report investigating heterogeneity (item 14).

In the low-quality classification, one systematic review^24^ received partial yes for the search strategy (item 4), did not justify the excluded primary studies (item 7), and did not describe the financial sources of included studies (item 10). The moderate quality reviews^25^, ^27^ did not perform items 3 and 10, and one did not perform data extraction in duplicate (item 6). The review classified as high quality^28^ failed only item 10.

A detailed analysis of the methodological quality assessment of the included systematic reviews is available in Table 3.

**Table 3:** Methodological quality assessment of included systematic reviews (AMSTAR-2).

Included systematic reviews	Bullous Diseases	1	2	3	4	5	6	7	8	9	10	11	12	13	14	15	16	Overall assessment
Asilian 2021^22^	BP	Y	N	N	N	Y	Y	N	Y	N	N	NA	NA	N	N	NA	Y	Critially Low quality
Atzmony 2014^23^	PV, PF	Y	N	N	N	Y	Y	N	Y	Y	N	Y	N	N	Y	N	Y	Critially Low quality
Atzmony 2015^24^	PV, PF	Y	Y	N	PY	Y	Y	N	Y	Y	N	Y	Y	Y	Y	Y	Y	Low quality
Kirtschig 2010^25^	BP	Y	Y	N	Y	Y	Y	Y	Y	Y	N	NA	NA	Y	Y	NA	Y	Moderate quality
Garcia-Doval 2013^26^	EB	Y	N	N	PY	N	N	N	Y	PY	N	NA	NA	N	N	NA	Y	Critially Low quality
Majumdar 2002^27^	TEN	Y	Y	N	PY	Y	N	Y	Y	Y	N	NA	NA	Y	Y	NA	Y	Moderate quality
Martin 2009^28^	PV, PF	Y	Y	Y	Y	Y	Y	Y	Y	Y	N	Y	Y	Y	Y	Y	Y	High quality
Singh 2011^29^	PV, PF, BP	Y	N	N	PY	N	N	N	Y	Y	N	NA	NA	N	N	NA	N	Critially Low quality
Zhao 2015^30^	PV	Y	N	N	PY	N	N	N	Y	N	N	NA	NA	N	N	NA	Y	Critially Low quality

Y, Yes; N, No; PY, Partially Yes; NA, Not Applied (no meta-analysis conducted). (http://amstar.ca/Amstar_Checklist.ph); BP, Bulloys Pemphigoid; PV, Pemphigus Vulgaris; PF, Pemphigus Foliaceus; EB, Epidermolysis Bullosa; TEN, Toxic Epidermal Necrolysis.

### Adequacy and quality of search strategies report

**Figure 3:**
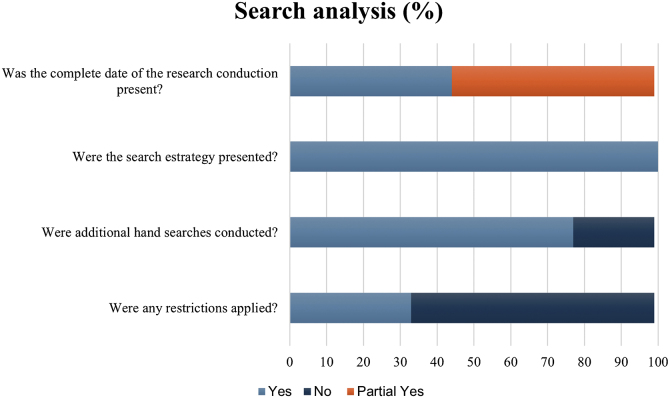
Additional analysis on search strategies of the included systematic reviews.

To assess the suitability of the search strategies presented by the included systematic reviews, the authors evaluated the data using five questions not comprised in the AMSTAR-2 tool. When checking the report of the search date, 44.4% (4/9) presented the complete date, and 55.5% (5/9) was partially full. All studies showed the databases' search strategies and 77.7% (7/9) performed additional hand searches. In 33.3 (3/9) studies, restrictions of language and/were applied. The most accessed databases were Cochrane Central Register of Controlled Trials (77.7%), MEDLINE (via Pubmed) (66.6%), Embase (55.5%), RCT register platforms (44.4%), LILACS (44.4%) and Cochrane Skin Group Specialised Register (33.3%) (Fig. 3).

### Number of systematic reviews that assessed the certainty of the evidence (GRADE)

All four 22, 23, 24, 29 reviews in which the GRADE approach could have been applied to assess the certainty of the body of evidence (as of the completion date and publication date of GRADE) did not.

### Number of systematic reviews that identify and report awaiting classification and ongoing studies

One review ^28^ identified and reported studies classified as 'awaiting classification' due to the absence of full-text articles, and seven reviews^23^, ^24^, ^25^, ^26^, ^27^, ^28^, ^30^ reported the ongoing RCTs identified after searching register platforms such as ClinicalTrials.gov.

### Overlapping RCTs in the included systematic reviews

Seven^22^, ^23^, ^24^, ^25^, ^28^, ^29^, ^30^ out of nine included systematic reviews (80%) that evaluated pemphigus and pemphigoid, giving 40 included RCTs on this condition. Of these, six^23^, ^24^, ^25^, ^28^, ^29^, ^30^ had at least two overlapping RCTs, with 65% (26/40) of the total RCTs in at least two reviews. The most recent review, published in 2021,^22^ included only three RCTs published between 2016 and 2017, whereas reviews published between 2009 and 2015 had at least nine RCTs published between 1988 and 2015, not identified by the first one.

## Discussion

Given the growing number of publications involving the treatment of vesiculobullous diseases, this meta-epidemiological study aimed to analyze the methodological quality of the systematic reviews published on this condition. Considering only the high level of evidence, nine systematic reviews of RCTs were identified, assessing different interventions for vesiculobullous skin diseases. Unfortunately, most were rated critically low and low quality according to the AMSTAR-2 criteria. Additionally, some reviews did not present adequate search strategy components and did not assess the certainty of the evidence through the GRADE approach and failed to identify and report awaiting classification and ongoing studies, as recommended by the Cochrane Handbook of Systematic Reviews of Interventions.^13^ Overlapping RCTs were also evaluated, simultaneously showing repeats in at least five reviews.

Using a tool such as AMSTAR-2 allows readers to identify the methodological quality of the evidence derived from a systematic review of randomized and non-randomized clinical trials, directly impacting the intended use of these results for clinical decision-making. It is essential to verify the meaning of the deviations committed by the review authors that were categorized as low or critically low quality.^11^

Systematic reviews are subject to bias; however, using a rigorous methodology allows researchers to access minimal systematic errors in their results.^13^ In this meta-epidemiological study, systematic reviews rated as low or critically low quality presented methodological weaknesses such as no report published register review protocol. The elaboration and publication of a previous review protocol reduce the possibility of reporting bias, that is when a study deviates from the methodological planning components and presents only selected outcomes that generally benefit the intervention of interest.^31^ Protocol drafting protects the results, even if they are harmful; any protocol differences justified in the published article also contribute to the reliability of the results.

None of the included systematic reviews explained the selection of study designs for the review, an important criterion when there are randomized clinical trials available on the research question, but there is a tendency to include non-randomized ones that may show incomplete summaries of the real treatment effects.^11^ Additionally, none of the included systematic reviews provided a list of excluded studies on the second selection stage with justifications, creating the risk that they remain invisible and the impact of their exclusion on the review unknown. The individual financing of the included RCTs in the systematic reviews was also not described by any review, and it could infer bias regarding financial conflict of interest.

The discussion and interpretation of the impact of the risk of bias of RCTs in the review findings are essential to support clinical decision-making, as is the analysis of these risks when pooling studies in meta-analysis. Heterogeneity between included RCTs should also be assessed through clinical question variation (PICO) and methodological considerations for having as homogeneous studies as possible.^11^

It was possible to observe a publication date interval between included systematic reviews. The newest review was published in 2021, classified as critically low quality, and did not include all available RCTs with the PICO question analyzed, included by older reviews. In addition, more than half of the included RCTs regarding pemphigus are duplicates between systematic reviews. This fact reflects the need to conduct practically simultaneous but methodologically heterogeneous systematic reviews, which may overestimate the intervention effects and confuse health professionals, policymakers, and patients. It is worth mentioning that one of the roles of a systematic review is to avoid duplication of effort by bringing together all published primary studies on an issue. This study observed that the critically low quality of several reviews duplicates unnecessary efforts and makes it unfeasible to use the findings to support clinical practice. In the end, despite the various reviews identified and several RCTs published, methodological rigor is still needed to clarify the best treatments for vesiculobullous diseases.

Furthermore, additional analysis regarding search strategies and the certainty of the evidence assessment using the GRADE approach is methodological criteria that could be incorporated into an updated version of the AMSTAR-2 tool, given the substantial impact these stages had on the results derived from a systematic review of interventions.

The present study has the advantage of conducting a comprehensive database search without any date or language restriction, making it difficult not to find published systematic reviews comprising the clinical question, and a rigorous methodology to select and critically appraise the included reviews, besides using a validated tool worldwide used to assess the methodological assessment of systematic reviews. To our knowledge, this is the first meta-epidemiological study to evaluate the quality of systematic reviews on treatment for vesiculobullous skin diseases.

There is a scarcity of systematic reviews with randomized clinical trials in some bullous dermatoses such as dermatitis herpetiformis, epidermolysis bullosa, erythema multiforme, Stevens-Johnson syndrome, bullous impetigo, hydroa vacciniforme, linear IgA bullous dermatosis. This is due to the low incidence of these diseases, which makes it challenging to design randomized clinical trials, the most appropriate study design for treatment questions. Therefore, the authors did not include systematic reviews that evaluated other study designs.

Regarding the included reviews, there are limitations related to methodological flaws and low quality of evidence. In addition, the AMSTAR-2 tool has some limitations related to its use; the tool was created in 2007 and updated in 2017, requiring adaptation and more time for application,^32^ and not as detailed a description of the search, requiring additional analysis to assess that.

## Conclusions

Considering the methodological quality of the systematic reviews on treatment for vesiculobullous diseases, 5 (55.6%) assessed bullous pemphigoid, epidermolysis bullosa, pemphigus vulgaris, and foliaceus were classified as critically low-quality. One review (11.1%) assessing pemphigus vulgaris and foliaceus was graded as high quality, 2 (22.2%) regarding toxic epidermal necrolysis and bullous pemphigoid as moderate quality, and 1 (11.1%) assessing pemphigus vulgaris and foliaceus was classified as low quality. Additionally, 26 randomized clinical trials overlapped in the included reviews, search strategies were not fully reported in most of them, and the GRADE approach was not used to assess the certainty of the evidence in any of the reviews. Therefore, there is an urgency to improve the methodological quality of future vesiculobullous disease systematic reviews to avoid unnecessary efforts and increase the reliability of this evidence to support the clinical decision.

## Financial support

None declared.

## Authors’ contributions

Kamilla Mayr Martins Sá: Conception and design of the study, data collection, article writing, acquisition, analysis, and interpretation of data, critical review of the literature, and final approval of the final version of the manuscript.

Juliana Cavaleiro Rodrigues: Acquisition, analysis, and interpretation of data, critical review of the literature, final approval of the final version of the manuscript.

Lígia Borges da Silva: Acquisition, analysis, and interpretation of data, critical review of the literature, final approval of the final version of the manuscript.

Giovanna Marcılio Santos: A critical review of the literature, critical review of important intellectual content, and final approval of the final version of the manuscript.

Mileny Esbravatti Stephano Colovati: A critical review of the literature, critical review of important intellectual content, final approval of the final version of the manuscript.

Ana Luiza Cabrera Martimbianco: Conception and design of the study, effective participation in the orientation of the research, article writing and critical review of important intellectual content, acquisition, analysis, and interpretation of data, critical review of the literature, final approval of the final version of the manuscript.

## Conflicts of interest

None declared.
